# Impact of Diet Quality during Pregnancy on Gestational Weight Gain and Selected Adipokines—Results of a German Cross-Sectional Study

**DOI:** 10.3390/nu14071515

**Published:** 2022-04-05

**Authors:** Christina Ehrhardt, Clara Deibert, Anne Flöck, Waltraut M. Merz, Ulrich Gembruch, Adeline Bockler, Jörg Dötsch, Christine Joisten, Nina Ferrari

**Affiliations:** 1 Department for Physical Activity in Public Health, Institute of Movement and Neurosciences, German Sport University Cologne, Am Sportpark Müngersdorf 6, 50933 Cologne, Germany; c.joisten@dshs-koeln.de; 2 Department of Pediatric, DRK Hospital Kirchen, Bahnhofstraße 24, 57548 Kirchen, Germany; clara_deibert@web.de; 3 Department of Obstetrics and Prenatal Medicine, Venusberg-Campus 1, University Bonn Medical School, 53127 Bonn, Germany; anne.floeck@ukb.uni-bonn.de (A.F.); waltraut.merz@ukb.uni-bonn.de (W.M.M.); ulrich.gembruch@ukb.uni-bonn.de (U.G.); 4 Department of General Internal Medicine/Cardiology, Marienhof Hospital, Rudolf-Virchow-Str. 7–9, 56073 Koblenz, Germany; bockler.adeline@gmail.com; 5 Department of Pediatrics and Adolescent Medicine, Faculty of Medicine, University Hospital Cologne and University of Cologne, Robert-Koch-Str. 16, 50931 Cologne, Germany; joerg.doetsch@uk-koeln.de; 6 Cologne Center for Prevention in Childhood and Youth/Heart Center Cologne, University Hospital of Cologne, Kerpener Str. 62, 50937 Cologne, Germany; nina.ferrari@uk-koeln.de

**Keywords:** pregnancy, nutrition, diet quality, gestational weight gain, adipokines

## Abstract

While nutrition during pregnancy is critical for the health of both mother and child, little is known about the diet quality of women during pregnancy, its correlation with gestational weight gain (GWG)/body composition, and chosen maternal adipokines. Therefore, we evaluated the Healthy Eating Index (HEI) of 110 pregnant women and analyzed its correlation with GWG/body composition, physical activity, leptin, resistin, adiponectin, and interleukin 6 (IL-6), respectively. Diet quality was medium in 63% of women, characterized by a high intake of animal-based products. HEI was negatively influenced by pre-pregnancy obesity (β = −0.335, *p* = 0.004), and positively influenced by higher age (>35 yrs., β = 0.365, *p ≤* 0.001), upper arm circumference (β = 0.222, *p* = 0.052), and total activity during the third trimester (β = 0.258, *p* = 0.008). GWG was associated with pre-pregnancy obesity (β = −0.512, *p ≤* 0.001), thigh circumference (β = 0.342, *p* = 0.007), upper arm fat area (β = 0.208, *p* = 0.092), and maternal age group (>35 yrs. β = −0.166, *p* = 0.082), but not with HEI. Leptin and IL-6 displayed associations with variables representative of body composition, such as pre-pregnancy BMI, thigh circumference, upper arm fat area, and upper arm circumference, but were not influenced by HEI. Neither were adiponectin and resistin. IL-6 was also associated with total activity. In conclusion, GWG, leptin, and IL-6 were influenced by nutritional status (body composition/pre-pregnancy BMI), not by maternal diet. Physical activity level also had an impact on IL-6. Thus, efforts should be intensified to improve diet quality and participation in sports before and during pregnancy, particularly in overweight or obese women.

## 1. Introduction

According to the Developmental Origins of Health and Disease (DOHaD) hypothesis, the first 1000 days from conception until the end of the second year of life are of particular importance in shaping health and susceptibility to chronic diseases in later life. An unhealthy maternal diet and a lack of physical activity result in permanent unfavorable alterations to structural and metabolic functions of the fetus by influencing endocrine programming, organ development, and epigenetic programming of gene expression [1]. Additionally, an imbalanced lifestyle may lead to excessive gestational weight gain (GWG) carrying a further risk of pregnancy and neonatal complications such as gestational diabetes mellitus (GDM), gestational hypertension, preterm birth, and large gestational age at birth [2,3].

Excessive GWG is mainly determined by an imbalance between energy intake and energy expenditure. Pregnant women often overestimate their energy needs, while underrating the importance of physical activity during gestation [4,5,6].

Sedentary behavior in combination with an increased energy intake is of particular concern, in that a rising number of women enter pregnancy already being overweight or obese. About 20% of German women of childbearing age are overweight, and another 9–14% are obese [7,8]. Similarly to excessive GWG, pre-pregnancy overweight and obesity have also been associated with an increased risk of adverse outcomes including GDM, hypertensive disorders, pre-term birth, and cesarean section [9]. Moreover, children born to obese mothers are more likely to develop childhood obesity and are at increased risk of long-term metabolic and behavioral consequences [9,10,11].

Although the mechanisms linking maternal obesity and excessive GWG with adverse pregnancy outcomes remain elusive, changes in maternal adipokine release owing to adipose tissue accumulation are thought to influence the metabolic environment [12]. Being critical signaling molecules, adipokines link maternal adipose tissue metabolism and nutritional status to placental development and function across gestation. They are involved in modulating placental nutrient transport and thus, may impact fetal development and growth [9]. Compared to normal weight pregnant women, obese women show increased levels of leptin and reduced levels of adiponectin. Both adipokines play important roles in metabolism and energy homeostasis. Additionally, maternal obesity has been reported to exacerbate the mild pro-inflammatory state associated with pregnancy [12].

Lifestyle factors such as diet and physical activity are known to modulate both BMI and GWG, but may also have a direct impact on maternal adipokines [13]. Preliminary results indicate a beneficial effect of regular physical activity on leptin sensitivity [14], plasma leptin levels [15,16,17], as well as IL-6 concentrations [15,18] for example. Furthermore, a healthy diet may contribute to attenuating the decrease in adiponectin levels during gestation [19,20] and has been recognized as an important modulator of chronic inflammation [21].

Owing to the importance of maternal nutritional status to prevent metabolic dysfunction and thus support the health of mother and child, health authorities have released diet and lifestyle recommendations to assist health care professionals in counseling women and young families with uniform, scientifically sound, and practical information [22,23]. However, it is not well-known yet whether the diet of pregnant women in Germany is in line with recommendations, and whether the quality of their diet impacts GWG and the concentration of selected adipokines.

Therefore, the aim of this cross-sectional study was to evaluate the diet quality of a cohort of pregnant women admitted for delivery at the Department of Obstetrics and Gynecology, University Hospital Bonn, and to analyze its correlation with GWG as well as maternal levels of the adipokines leptin, resistin, adiponectin, and IL-6.

## 2. Materials and Methods

This cross-sectional cohort study conducted from December 2013 to April 2014 at the Department of Obstetrics and Gynecology, University Hospital Bonn, Germany included 123 pregnant women admitted for delivery at a gestational age between 36 and 42 weeks. Exclusion criteria comprised gestational age < 36 weeks, multiple pregnancies, mental illness, or prenatally detected malformations. Women who were not able to speak German were also excluded.

Ethical approval for this study was obtained from the institutional ethics committee (reference number: 269/13). The study was conducted in accordance with the ethical principles of medical research on humans (Declaration of Helsinki) and the World Medical Association. All study participants signed informed consent forms affirming their voluntary participation.

### 2.1. Anthropometric, Demographic, and Clinical Data

The following participant information was retrieved from either medical files or health insurance cards: age, height, weight (before pregnancy and at admission for labor), parity, ethnicity, level of education, smoking behavior, mode of delivery, presence of gestational diabetes, presence of pre-eclampsia, and other complications during pregnancy (for details see [15,24,25]).

Pre-pregnancy body mass index (BMI) was calculated using the formula: body weight (kg)/(body height (m))^2^ and categorized into the following BMI categories: underweight (<18.5 kg/m^2^), normal weight (18.5–24.9 kg/m^2^), overweight (25.0–29.9 kg/m^2^), or obese (≥30.0 kg/m^2^) [26]. Weight changes during pregnancy were generated from medical files and were calculated using the difference between the weight measured at the most recent antenatal care visit and the weight before gestation. Additionally, GWG was classified according to international guidelines (Table 1; [27]).

In addition, two measurements for the assessment of body composition were obtained. The first was the upper arm and thigh circumference, on the right side, using a non-flexible measuring tape with an accuracy of 0.1 cm. Second, the skinfold thickness for various body parts, including the triceps, hips, front axillary line at the height of the tenth rib, and rectus femoris was evaluated using a Harpenden Skinfold Caliper (John Bull British Indicators Ltd., Harpenden, UK) with an accuracy of 0.2 mm and constant contact pressure (10 g/mm^2^). Each body part was measured three times, and a mean value was obtained. The upper arm and thigh fat mass were estimated based on the circumference of each limb and the mean skinfold thickness, using the following formula: UFE = C × (TS/2) and TUA = C2/(4π), where UFE is the upper arm/thigh fat area estimate, C is the upper arm/thigh circumference, TS is the triceps skinfold thickness, and TUA is the total upper arm [28].

### 2.2. Diet and Physical Activity

Dietary habits during pregnancy were documented once at admission for delivery by means of a semi-quantitative food frequency questionnaire (FFQ) adapted from a questionnaire described elsewhere [29]. The FFQ was designed to record the average habitual dietary intake during the entire pregnancy. The average frequency of consumption for various food categories was documented including fruits and vegetables, milk and dairy products, eggs, beverages (including coffee and alcohol), meat and meat products, fish and seafood, bread, grains and cereals, sweets and salty snacks as well as fats and oils. Frequency categories were used in increasing order (not at all, times per week or day) and amounts were given in standard portion sizes.

In addition, the use of dietary supplements was recorded and diet quality was assessed by the Healthy Eating Index (HEI)-NVS II as described by Hoffmann and Spiller 2010 [30]. HEI-NVS II comprises 10 categories (Supplementary Table S1); in each of them, a maximum score of 10 to 15 can be attained. The more the consumption corresponds to dietary recommendations [8,31], the higher the HEI. A total of 110 points can be attained by adding up the individual indices. An HEI of >80% of the maximum points was qualified as “good dietary quality”, >50 to 80% was regarded as “medium dietary quality”, and a score of <50% indicated a “low dietary quality”.

Physical activity was assessed by the Pregnancy Physical Activity Questionnaire (PPAQ) as described by Chasan-Taber et al. [32] and measured in metabolic equivalents (METs) [33] by multiplying the time spent in each activity by its intensity. According to the METs, the activity of each trimester was classified into the following intensity groups: sedentary (<1.5 METs), light (1.5–3.0 METs), moderate (3.0–6.0 METs), or vigorous (>6 METs).

### 2.3. Selected Laboratory Parameters

Upon submission to the labor ward, a non-fasting maternal venous blood sample (7.5 mL serum tube; S-Monovette, Sarstedt; Nümbrecht, Germany) was drawn. Blood samples were stored at a 0.4 °C for a maximumof 48 h and centrifuged (4000 rpm for 10 min at 4 °C, Hettich MR centrifuge; (Tuttlingen, Germany)). The serum was pipetted and moved to a new tube for storage (at −20 °C) until analysis. Leptin and adiponectin were quantified by a direct sandwich enzyme-linked immunosorbent assay (ELISA kit, Merck/Millipore KGaA, Darmstadt, Germany), according to the manufacturer’s instructions and using a TECAN reader (Nano Quant infinite M200 Pro, Männedorf, Switzerland). A seven-point standard curve was generated in each plate with a minimum level of detection of 0.78 ng/mL and 1.28 ng/mL for leptin and adiponectin, respectively. IL-6 and resistin levels were assessed by a multiplex immunoassay (eBioscience, San Diego, CA, USA) and read using a Luminex 200 reader (Luminex, Austin, TX, USA). A seven-point standard curve was generated on each plate, with minimum detection levels of 9.1 and 6.01 pg/mL for IL-6 and resistin, respectively (calculated with Bio-Plex Manager 6.1, Bio-Rad, Hercules, CA, USA).

### 2.4. Data Analysis and Statistics

Statistical analysis was performed using the SPSS statistical package (IBM SPSS Statistics for Windows, Version 27, IBM Corp., Armonk, NY, USA). Descriptive statistics were applied to present anthropometric and lifestyle data. Mean values and standard deviations were calculated and significance was defined as a *p*-value of ≤0.05. All confidence intervals (CIs) were estimated at the 95% level.

A *t*-test was performed for 2-group comparisons for metric variables (e.g., food intake, HEI) and χ^2^ test as well as a 2-sided Fisher test for categorical and dichotomous variables (e.g., a difference in diet quality over pre-pregnancy BMI groups, GWG groups, age groups). Backward multiple linear regression analysis was used to assess the individual factors influencing HEI, GWG, and adipokine levels. The variables “maternal age”, and “pre-pregnancy BMI” were included as dichotomous variables. Maternal age group was defined as 0 = 18–34 years, and 1 = 35 years and older (risky pregnancy). Pre-pregnancy BMI classes were set as 0 = BMI ≤ 29.9 kg/m^2^, 1 = BMI ≥ 30 kg/m^2^ (obese).

The initial model for HEI included the following variables: maternal age group, pre-pregnancy BMI class, total activity (TA) during the 1st, 2nd, and 3rd trimester (METs), parameters of body composition (thigh circumference, upper arm circumference, total upper arm area, and upper arm fat area), maternal education, marital status, and GWG.

The initial model for GWG included the variables maternal age group, pre-pregnancy BMI class, total activity (TA) during the 1st, 2nd, and 3rd trimester (METs), parameters of body composition (thigh circumference, upper arm circumference, total upper arm area, and upper arm fat area), maternal education, marital status, and HEI (total).

For leptin, adiponectin, resistin, and IL-6 following variables were chosen: maternal age group, pre-pregnancy BMI class, TA during the 1st–3rd trimester (METs), parameters of body composition (thigh circumference, upper arm circumference, total upper arm area, and upper arm fat area), and HEI (total).

The number of cases may vary in the following results section. In some cases, the evaluated blood parameters were below the detection limit and were therefore excluded from the analysis. In other cases, the questionnaire was not completely filled out, or the measurement of body composition could not be performed.

## 3. Results

**Figure 1:**
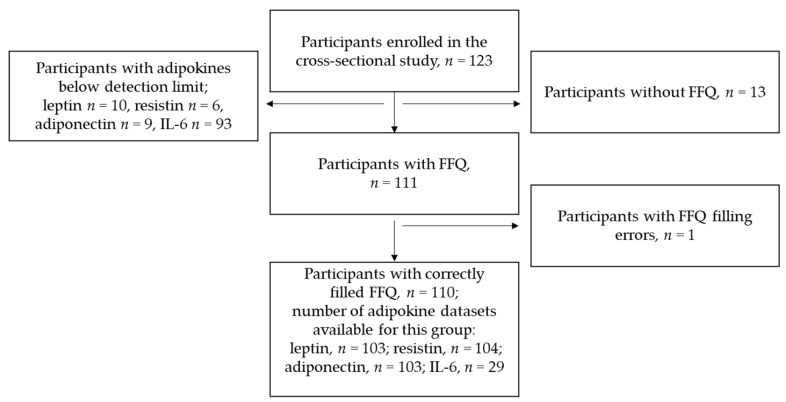
Flow chart of the sample included in the study process; FFQ, food frequency questionnaire.

Of the 123 women included in the study, 111 completed the FFQ. One woman was excluded from dietary analyses because the calculated energy intake based on the FFQ was almost 15,000 kcal/day, which appeared to be unrealistic, and rather indicated a mistake in filling in the questionnaire. Thus, data of 110 women were included in the analysis of dietary quality (Figure 1). Some of these women had adipokine levels below the level of detection. The exact number of data sets available for each adipokine is displayed in Figure 1.

### 3.1. Characteristics of the Study Population

**Figure 2:**
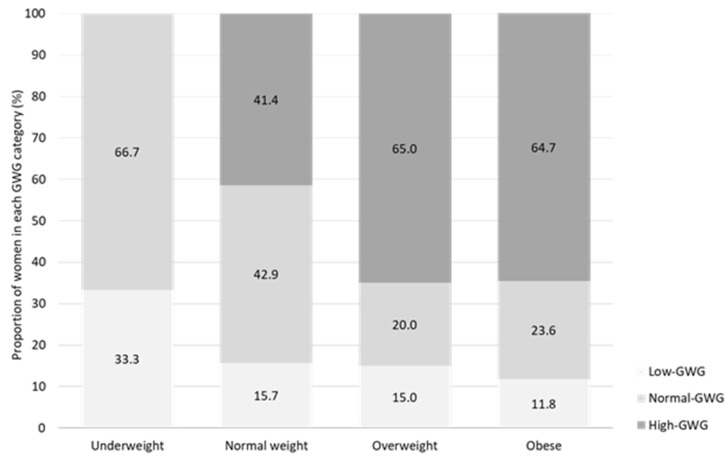
Weight gain according to IOM guidelines [27] depending on weight classification. GWG, gestational weight gain.

Women participating in our study were 33.5 ± 4.6 years old and had a mean pre-pregnancy BMI of 24.9 ± 5.9 kg/m^2^. The majority of women were normal weight (63.6%), while 18.2% and 15.5% were overweight and obese, respectively (Table 2). The mean GWG was 15.3 ± 6.5 kg. A GWG within the current recommendations of the Institute of Medicine (Normal-GWG) was achieved by 36.4% of women, and about every second woman exceeded the recommendations (48.2%, High-GWG; Table 2). The proportion of women who gained more weight than recommended was particularly high in women entering pregnancy being overweight or obese (Figure 2). Further participants’ characteristics are presented in Table 2.

### 3.2. Diet during Pregnancy

The majority of women followed an omnivorous diet including meat and fish (78.9%). Consuming meat, but no fish was stated by 11.9%, whereas 4.6% ate fish, but no meat. Only a minority of women ate a vegetarian diet (ovo-lacto-vegetarian 3.7%, lacto-vegetarian 0.9%).

#### 3.2.1. Actual Food Intake and Comparison with Dietary Recommendations

Comparing the actual food intake with the nutrition guidelines of the German Society for nutrition, overconsumption was observed for the intake of meat/meat products, milk/milk products, fruits, vegetables, and spreadable fats, (Table 3), with no significant differences between pre-pregnancy BMI, GWG or age groups (data not shown). The mean consumption of grains and eggs was within the recommended ranges. However, only 38.2% of women achieved the intake recommendations for grains, while every second woman consumed less than the recommended amounts. Moreover, one-third (32.7%, incl. 3.6% vegetarians) did not meet the recommendation for fish, as they ate less than one serving of fish per week or no fish at all (Table 3).

#### 3.2.2. Dietary Supplement Intake, Alcohol Intake, Smoking Status

Eighty-eight percent of women took dietary supplements during pregnancy, whereas 12.0% did not use any supplements. Among women taking supplements, all followed a daily (6–7 times/week) routine of folic acid, B vitamins, or multivitamin intake. Regular supplementation (6–7 times/week) of iron (37.0%), fish oil (17.8%), iodine (20.4%), calcium (15.0%), or magnesium (40.6%) was documented. As to smoking habits, 93.6% of participants were non-smokers, and 6.4% smoked regularly. None of the women drank alcohol during pregnancy.

### 3.3. Diet Quality

Mean HEI was 83.4 ± 11.5. Diet quality was medium (50–80% of max total HEI points) in 62.7% of women and good (> 80% of max total HEI points) in 37.3% of women, respectively with no significant differences over GWG groups (Chi^2^ *p* = 0.315) or pre-pregnancy BMI categories (Chi^2^ *p* = 0.314). However, the proportion of women with “good” and “medium” diet quality differed over age groups (2-sided Fisher test: *p* = 0.028). Fifty percent of women aged ≥ 35 years followed a “good” quality diet, compared to only 28.1% in the group of women aged 18–34 years.

#### 3.3.1. Characteristics of “Medium” and “Good” Diets

Compared to women following a “high quality” diet, those eating a “medium quality” diet consumed significantly more milk and dairy including cheese (879.71 g/day vs. 664.6 g/day, *p* = 0.022), red meat and meat products (970.9 g/week vs. 602.2 g/week, *p* = 0.004), spreadable fats (39.8 g/d vs. 21.8 g/day, *p* = 0.001) as well as less fruits (319.8 vs. 408.6 g/day, *p* = 0.044) and vegetables (645.7 vs. 995.1 g/day, *p* ≤ 0.001) (for further information see Supplementary Table S2).

#### 3.3.2. Comparison of HEI between GWG-, BMI-, and Age Groups

Comparing HEI or single HEI categories between GWG groups, no significant differences were present, except for the HEI category “fruits”, which was significantly higher in the High-GWG group compared to the Normal-GWG group (12.1 ± 3.6 vs. 10.0 ± 4.5, *p* = 0.015; Supplementary Table S3). Assessment of diet quality by pre-pregnancy BMI class found a significantly lower HEI in obese women compared to women with a BMI < 30 kg/m^2^ (77.8 ± 11.5 vs. 84.5 ± 11.3, *p* = 0.027; Supplementary Table S4). A closer look at HEI categories revealed significant differences for the HEI categories “beverages” (9.4 ± 2.1 vs. 10.0 ± 0.2, *p* = 0.020) and “fish” (2.9 ± 3.4 vs. 5.4 ± 4.1, *p* = 0.020), as well as a trend for the HEI category “fruits” (9.6 ± 5.0 vs. 11.5 ± 3.9, *p* = 0.068, Supplementary Table S4).

Comparing the diet quality of women in different age groups, women aged 18–34 years had a significantly lower HEI than women 35 years and older (80.7 ± 11.5 vs. 87.3 ± 10.6, *p* = 0.002). Particularly, the HEI category “vegetables” showed significant differences (11.4 ± 4.4 vs. 13.6 ± 2.7, *p* = 0.004; Supplementary Table S5).

#### 3.3.3. Factors Associated with HEI

Multiple linear regression analysis was performed to evaluate factors that are associated with diet quality (HEI, total). In the final model, we found maternal age group (>35 years: β = 0.365, *p ≤* 0.001), pre-pregnancy BMI class (obesity: β = −0.335, *p* = 0.004), upper arm circumference (β = 0.222, *p* = 0.053) as well as total activity during the 3rd trimester (β = 0.258, *p* = 0.008) to be significantly associated with HEI. These variables explained 24.6% of the HEI variance. Further details can be found in Table 4.

### 3.4. Factors Associated with Gestational Weight Gain (GWG)

Multiple linear regression analysis was performed to assess the factors influencing GWG. While diet quality (HEI, total) was not associated with GWG, pre-pregnancy BMI class (obesity: β = −0.512, *p ≤* 0.001), thigh circumference (β = 0.342, *p* = 0.007), upper arm fat area (β = 0.208, *p* = 0.092), and maternal education (β = −0.166, *p* = 0.082) explained 20.2% of the variation in GWG (Table 5).

### 3.5. Factors Associated with Selected Adipokines

Leptin was associated with pre-pregnancy BMI class (obesity β = 0.227, *p* = 0.046), and markers of body composition, namely thigh circumference (β = 0.278, *p* = 0.017) and upper arm fat area (β = 0.212, *p* = 0.060); diet quality had no impact on leptin. In the final model, these factors contributed to 37.5% of the leptin variance (Table 6).

Resistin was not found to be associated with any of the variables investigated (data not shown), while adiponectin displayed a non-significant association with GWG (β = 0.192, *p* = 0.067; Table 7).

IL-6 was influenced by thigh circumference (β = 1.098, *p ≤* 0.001), upper arm circumference (β = −1.820, *p* ≤ 0.001), upper arm fat area (β = 0.951, *p* = 0.007), and total activity during the 2nd trimester (β = −0.389, *p* = 0.019, Table 8).

## 4. Discussion

To the best of our knowledge, this is the first study analyzing the association of diet quality with GWG and adipokines in a cohort of pregnant women in Germany. Only one-third of our participants followed a healthy-balanced diet as recommended by Nutrition and Health Authorities. In particular, intakes of animal-based products such as meat/meat products and milk/milk products exceeded recommendations. Additionally, a high proportion of women exceeded GWG recommendations. Obese and younger women scored significantly lower on the total HEI than elder women and those with a BMI < 30 kg/m^2^. Considering the cross-sectional design, neither GWG nor selected adipokines were influenced by diet quality, but nutritional status (pre-pregnancy BMI/body composition) was significantly associated with GWG and leptin as well as IL-6.

Although the importance of a healthy diet before and during pregnancy is well established, little is known about the relationship between prenatal diet quality with excessive GWG and available evidence has been inconsistent [35,36,37,38]. Similar to other studies in healthy pregnant women [36,39], HEI did not have an impact on GWG. Although diet quality scores such as the HEI as used in our study may provide useful information on total diet, they are based primarily on subjective information. Therefore, diet quality scores are prone to bias, which may have impacted our analysis.

In contrast to diet quality, parameters representative of nutritional status (e.g., pre-pregnancy BMI) had an impact on GWG. Being obese was associated with a lower GWG during pregnancy compared to women with a pre-pregnancy BMI < 30 kg/m^2^. Additionally, we observed a positive association between pre-pregnancy obesity and leptin. Markers of maternal body composition such as thigh circumference and upper arm fat mass were positively associated with IL-6. In contrast, physical activity during the 2nd trimester is inversely correlated with IL-6. The benefits of physical activity are well established. It is associated with reduced risk for obesity and excessive GWG, improved psychologic wellbeing, and lower maternal systemic inflammation [15,40]. However, in contrast to recommendations, the majority of pregnant women in Germany and other countries have a low level of physical activity and rather reduce their participation in sports during pregnancy or do not exercise at all [6,41]. According to a survey, 80% of pregnant women did no exercise or had reduced their participation in sports. Only 20% reported engaging in the same amount or more sports than in the pre-pregnant state [42]. In our cohort, only 34.5% of women achieved the current recommendations for sports of at least 150 min of moderate-intensity aerobic physical activity throughout the week (data not shown) [43]. Similarly, international data indicate that pregnant women spend much of their time in sedentary activities [44].

Therefore, individual counselling in terms of diet quality and physical activity before and during pregnancy may provide significant health benefits for mother and child, albeit further research is needed to shed light on the exact mechanisms involved in linking diet to maternal metabolic dysfunction and in turn, offspring’s later risk for metabolic diseases. 

### Limitations of the Study

A major limitation of this study is the self-reported data on dietary intake, which is prone to systematic bias. Filling in dietary records such as FFQs, study participants regularly tend to answer questions in a way that will be viewed favorably by others involving an overreporting of healthy dietary choices such as fruit and vegetable intake [45] and an underreporting of unhealthy habits [46]. Additionally, when recalling the usual diet over a long time such as pregnancy, accuracy and precision can be limited and the indicated frequency of consumption and portion size may not represent the usual intake of respondents. FFQs also require certain literacy skills and depends on the ability to describe the diet [47]. Moreover, dietary intake was only obtained once, on the day of admission for delivery, which may not be representative of the diet followed throughout pregnancy. The HEI used to assess diet quality also has some limitations, as it does not take into account the exact quality of various foods in specific groups such as beverages (e.g., sweetened vs. unsweetened) and grains (e.g., whole grain or refined products), and was not specifically designed for pregnant women.

Furthermore, our cohort may not be representative of the general population, because participants were recruited only from the obstetric unit of the University Bonn Medical School. Although we performed consecutive recruitment to avoid potential selection bias, the educational status of our population was quite high and the increased occurrence of specific subpopulations, such as increased interest in study participation among health-conscious women, cannot be ruled out.

A further limitation of the present work is the cross-sectional nature of the study design, which generally excludes the assessment of cause-effect relationships. Moreover, the sample size may not have been sufficient to shed light on potential associations between diet quality and GWG or adipokines. Particularly, the number of datasets available for the analysis of IL-6 was small and the results need to be interpreted with caution. The fact that we obtained the blood samples only once upon admission for delivery may further limit the representativity of adipokine concentrations.

The performance of all investigations according to standardized procedures and the standardized sample processing served to prevent an information bias. Nevertheless, larger studies are warranted to confirm our findings.

## 5. Conclusions

Nutritional status and diet quality before and during gestation may not only impact GWG but also levels of selected maternal adipokines. However, the moderate dietary quality and high rate of excessive GWG observed in our study confirmed previous observations that many pregnant women fall short in complying with recommendations for diet and physical activity. Reasons for a low guideline adherence may be insufficient knowledge and/or understanding of the specific recommendations and the skills to put them in use [48,49].

In order to improve diet quality and encourage physical activity, lifestyle advice may be intensified towards personalized guidance followed up during gestation. Pregnant women have recently been reported to perceive tailored dietary counseling as critical to achieving the implementation of healthier nutrition. Additionally, the first evidence suggested that personalized dietary interventions may be a successful approach to limiting GWG [50] and improving diet quality in adults [51]. Identification of barriers and enablers to change as well as the inclusion of practical support (e.g., cooking classes, stress reduction practices) seem to be important aspects to achieve a long-term improvement of diet quality [52,53,54,55]. Similarly, clearer guidance and personal education on behavioral change techniques have been helpful in improving physical activity during pregnancy [56,57].

Since health practitioners often lack time and sufficient knowledge to provide adequate counseling, a collaboration with dieticians, nutritionists, sports scientists, and other health care professionals appears indispensable. The use of validated digital tools may further support the promotion of healthy diet behaviors [58] and physical activity, particularly in younger women or those with limited access to personal support. In parallel, the creation of professionally curated online platforms may be meaningful to limit exposure to potentially misleading information and impart useful knowledge to women [57].

## Figures and Tables

**Table 1:** GWG recommendations from the 2009 Institute of Medicine and National Research Council Report [27].

Pre-Pregnancy BMI Category	Total Weight Gain
Underweight (<18.5 kg/m^2^)	12.5–18 kg (28–40 lbs)
Normal weight (18.5–24.9 kg/m^2^)	11.5–16 kg (25–35 lbs)
Overweight (25.0–29.9 kg/m^2^)	7–11 kg (15–25 lbs)
Obesity (≥30.0 kg/m^2^)	5–9 kg (11–20 lbs)

BMI, Body Mass Index; GWG, gestational weight gain.

**Table 2:** Participants’ characteristics (mean ± SD/*n* (%)).

Parameter	*n*	Mean ± SD/*n* (%)	Minimum	Maximum
Age (years)	110	33.5 ± 4.6	18.0	43.6
35 years and older		46 (41.8%)		
Pre-pregnancy weight (kg)	110	70.8 ± 16.9	48.0	146.0
Pre-pregnancy BMI (kg/m^2^)	110	24.9 ± 5.9	17.1	50.0
Weight before delivery (kg)	110	86.5 ± 16.7	57.4	150.2
Gestational weight gain (kg)	110	15.3 ± 6.5	1.6	45.7
Upper arm circumference (cm)	105	27.7 ± 3.8	18.0	41.0
Thigh circumference (cm)	105	51.3 ± 7.5	35.0	73.0
Total upper arm area (cm^2^)	104	61.9 ± 17.8	25.8	133.8
Upper arm fat area (cm^2^)	104	31.2 ± 14.6	7.4	106.1
Pre-pregnancy BMI classes	110			
Underweight (<18.5 kg/m^2^)		3 (2.7%)		
Normal weight (18.5–24.9 kg/m^2^)		70 (63.6%)		
Overweight (25.0–29.9 kg/m^2^)		20 (18.2%)		
Obese (≥30 kg/m^2^)		17 (15.5%)		
Gestational weight gain classes	110			
Below IOM recommendation		17 (15.5%)		
Within IOM recommendation		40 (36.4%)		
Above IOM recommendation		53 (48.2%)		
Smoking	109	7 (6.4%)		
Gestational diabetes mellitus	107	18 (16.8%)		
Married	110	85 (77.3%)		
Higher education (A-level)	109	86 (78.9%)		
Nationality German	106	97 (91.5%)		
Physical activity				
Before pregnancy (METs)	109	376.6 ± 177.0	132.5	929.0
1st trimester (METs)	109	348.3 ± 171.6	0.0	896.5
2nd trimester (METs)	109	317.7 ± 159.5	0.0	892.0
3rd trimester (METs)	109	269.4 ± 132.5	33.0	869.7
Diet quality				
HEI, total	110	83.4 ± 11.5	58.4	108.8
Good diet quality		41 (37.7%)		
Medium diet quality		69 (62.7%)		
Biochemistry				
Leptin (ng/mL)	103	23.6 ± 18.3	1.0	83.3
Resistin (pg/mL)	104	9137.3 ± 3740.4	3068.2	27,600.9
Adiponectin (ng/mL)	103	21.4 ± 11.9	5.6	84.3
IL-6 (ng/mL)	29	32.4 ± 27.2	9.7	147.2

BMI, Body Mass Index; GWG, gestational weight gain; IL-6, interleukin-6; IOM, Institute of Medicine; METs, metabolic equivalents.

**Table 3:** Recommendations for dietary intake [31,34] and actual intake by participants.

Food Category	Recommended Intake	Mean ± SD	Min.	Max.	Correspondence with Recommendations		
					Yes	More	Less
Grains (g/day)	350–550	372.1 ± 164.0	99.3	960.1	38.2%	10.0%	51.8%
Fruits (g/day)	250	352.84 ± 224.1	0.0	1000.0	76.4%	-	23.6%
Vegetables (g/day)	400	775.9 ± 468.7	0.0	2325.0	63.6%	-	36.4%
Milk, milk products (g/day)	100–500	799.6 ± 470.2	0.0	2700.0	34.5%	60.9%	4.5%
Meat, meat products (g/week)	<300–500	938.9 ± 494.8	0.0	3070.0	26.4%	73.6%	0.0%
Eggs (number/week)	<3	2.1 ± 1.4	0.0	7.0	74.5%	73.6%	-
Fish (g/week)	150–220	231.8 ± 264.6	0.0	1350.0	49.1%	18.2%	32.7%
Spread (g/day)	<15–30	33.1 ± 28.7	0.0	148.6	60.0%	40.0%	-
Beverages (ml/week)	>1500 ml	2934.6 ± 1294.4	200	8400.0	96.4%	-	3.6%

**Table 4:** Multiple linear regression: Diet quality (HEI, total) as an outcome variable (baseline and final model, *n* = 98).

Models	Variable	B	SE	β-Coefficient	*p*-Value	*R* ^2^
Baseline	Maternal age group	8.453	2.385	0.360	≤0.001	0.260
	Gestational weight gain (kg)	−0.022	0.194	−0.012	0.910	
	Pre-pregnancy BMI class	−10.169	4.161	−0.334	0.017	
	Thigh circumference (cm)	0.089	0.244	0.058	0.717	
	Upper arm circumference (cm)	0.515	2.866	0.169	0.858	
	Total upper arm area (cm^2^)	0.042	0.651	0.065	0.948	
	Upper arm fat area (cm^2^)	−0.065	0.152	−0.083	0.672	
	Total activity, 1st trimester (METs)	−0.002	0.011	−0.030	0.848	
	Total activity, 2nd trimester (METs)	0.012	0.015	0.171	0.402	
	Total activity, 3rd trimester (METs)	0.012	0.014	0.139	0.396	
	Marital status	1.072	2.027	0.053	0.598	
	Maternal education	0.376	2.200	0.018	0.865	
Final	Maternal age group	8.576	2.205	0.365	≤0.001	0.246
	Upper arm circumference (cm)	0.677	0.346	0.222	0.053	
	Pre-pregnancy BMI class	−10.189	0.008	−0.335	0.004	
	Total activity, 3rd trimester (METs)	0.023	0.008	0.258	0.008	

B, regression coefficient B; β-coefficient, standardized coefficient beta; BMI, Body Mass Index; METs, metabolic equivalents; HEI, Healthy Eating Index; SE, standard error.

**Table 5:** Multiple linear regression: gestational weight gain (GWG) as an outcome variable (baseline and final model, *n* = 98).

Models	Variable	B	SE	β-Coefficient	*p*-Value	*R* ^2^
Baseline	Maternal age group	0.542	1.429	0.042	0.706	0.230
	Pre-pregnancy BMI class	−8.459	2.228	−0.506	≤0.001	
	Thigh circumference (cm)	0.353	0.131	0.423	0.009	
	Upper arm circumference (cm)	0.593	1.603	0.355	0.712	
	Total upper arm area (cm^2^)	−0.193	0.364	−0.543	0.589	
	Upper arm fat area (cm^2^)	0.142	0.084	0.333	0.094	
	Total activity, 1st trimester (METs)	−0.005	0.006	−0.129	0.424	
	Total activity, 2nd trimester (METs)	0.002	0.008	0.055	0.792	
	Total activity, 3rd trimester (METs)	0.007	0.008	0.149	0.899	
	HEI, total (points)	−0.007	0.061	−0.013	0.910	
	Marital status	−0.074	1.136	−0.007	0.948	
	Maternal education	−2.345	1.205	−0.209	0.055	
Final	Pre-pregnancy BMI class	−8.551	2.041	−0.512	≤0.001	0.202
	Thigh circumference (cm)	0.285	0.103	0.342	0.007	
	Upper arm fat area (cm^2^)	0.089	0.052	0.208	0.092	
	Maternal education	−1.860	1.058	−0.166	0.082	

B, regression coefficient B; β-coefficient, standardized coefficient beta; BMI, Body Mass Index; METs, metabolic equivalents; HEI, Healthy Eating Index; SE, standard error.

**Table 6:** Multiple linear regression: Leptin as an outcome variable (baseline and final model, *n* = 92).

Models	Variable	B	SE	β-Coefficient	*p*-Value	*R* ^2^
Baseline	Maternal age group	−1.667	3.780	−0.044	0.660	0.410
	Gestational weight gain (kg)	0.238	0.276	0.083	0.391	
	Pre-pregnancy BMI class	12.953	6.274	0.269	0.042	
	Thigh circumference (cm)	0.493	0.361	0.203	0.175	
	Upper arm circumference (cm)	6.088	4.117	1.252	0.143	
	Total upper arm area (cm^2^)	−1.260	0.934	−1.223	0.181	
	Upper arm fat area (cm^2^)	0.247	0.221	0.201	0.266	
	Total activity, 1st trimester (METs)	0.003	0.014	0.028	0.838	
	Total activity, 2nd trimester (METs)	0.001	0.021	0.011	0.951	
	Total activity, 3rd trimester (METs)	−0.021	0.021	−0.153	0.311	
	HEI, total (points)	0.023	0.163	0.014	0.885	
Final	Pre-pregnancy BMI class	10.910	5.394	0.227	0.046	0.375
	Thigh circumference (cm)	0.677	0.279	0.278	0.017	
	Upper arm fat area (cm^2^)	0.260	0.137	0.212	0.060	

B, regression coefficient B; β-coefficient, standardized coefficient beta; BMI, Body Mass Index; METs, metabolic equivalents; HEI, Healthy Eating Index; SE, standard error.

**Table 7:** Multiple linear regression: Adiponectin as an outcome variable (baseline and final model, *n* = 92).

Models	Variable	B	SE	β-Coefficient	*p*-Value	*R* ^2^
Baseline	Maternal age group	4.102	3.053	0.165	0.183	0.104
	Gestational weight gain (kg)	0.224	0.223	0.119	0.318	
	Pre-pregnancy BMI class	−6.780	5.067	−0.218	0.179	
	Thigh circumference (cm)	0.221	0.291	0.139	0.450	
	Upper arm circumference (cm)	−0.776	3.325	−0.244	0.816	
	Total upper arm area (cm^2^)	0.007	0.754	0.011	0.992	
	Upper arm fat area (cm^2^)	0.187	0.178	0.232	0.298	
	Total activity, 1st trimester (METs)	−0.007	0.012	−0.099	0.554	
	Total activity, 2nd trimester (METs)	0.007	0.017	0.093	0.696	
	Total activity, 3rd trimester (METs)	0.007	0.017	0.072	0.696	
	HEI, total (points)	−0.020	0.131	−0.018	0.882	
Final	Gestational weight gain (kg)	0.362	0.195	0.192	0.067	0.037

B, regression coefficient B; β-coefficient, standardized coefficient beta; BMI, Body Mass Index; METs, metabolic equivalents; HEI, Healthy Eating Index; SE, standard error.

**Table 8:** Multiple linear regression: IL-6 as an outcome variable (baseline and final model, *n* = 27).

Models	Variable	B	SE	β-Coefficient	*p*-Value	*R* ^2^
Baseline	Maternal age group	7.722	16.782	0.140	0.652	0.581
	Gestational weight gain (kg)	0.030	0.894	0.007	0.973	
	Pre-pregnancy BMI class	15.019	18.487	0.250	0.429	
	Thigh circumference (cm)	3.683	1.515	0.977	0.028	
	Upper arm circumference (cm)	−6.979	13.662	−1.074	0.617	
	Total upper arm area (cm^2^)	−1.101	3.176	−0.834	0.734	
	Upper arm fat area (cm^2^)	1.449	0.852	0.973	0.110	
	Total activity, 1st trimester (METs)	0.022	0.054	0.142	0.692	
	Total activity, 2nd trimester (METs)	−0.082	0.080	−0.479	0.321	
	Total activity, 3rd trimester (METs)	0.030	0.087	0.125	0.732	
	HEI, total (points)	−0.452	0.516	−0.217	0.395	
Final	Thigh circumference (cm)	4.139	1.051	1.098	≤0.001	0.512
	Upper arm circumference (cm)	−11.830	2.861	−1.820	≤0.001	
	Upper arm fat area (cm^2^)	1.426	0.476	0.951	0.007	
	Total activity, 2nd trimester (METs)	−0.066	0.026	−0.389	0.019	

B, regression coefficient B; β-coefficient, standardized coefficient beta; BMI, Body Mass Index; METs, metabolic equivalents; HEI, Healthy Eating Index; SE, standard error.

## Data Availability

Not applicable.

## Supplementary Materials

The following supporting information can be downloaded at: https://www.mdpi.com/article/10.3390/nu14071515/s1, Table S1: Components of the HEI-NVS and their standards for scoring adapted from [30], Table S2: Dietary intake of women following a “good quality” or “medium quality” diet, Table S3: Comparison of the Healthy Eating Index (HEI, total and subcategories) by GWG groups, Table S4:Comparison of the Healthy Eating Index (HEI, total and subcategories) by pre-pregnancy class, Table S5: Comparison of the Healthy Eating Index (HEI, total and subcategories) by age groups.
