# Supplementary material for: Impact of Diet Quality during Pregnancy on Gestational Weight Gain and Selected Adipokines—Results of a German Cross-Sectional Study
Source: Nutrients. 2022 Apr 5;14(7):1515. doi: 10.3390/nu14071515 (PMC9003101; doi:10.3390/nu14071515)
Supplement: Supplementary file 1 [file nutrients-14-01515-s001.zip › nutrients-1625433-supplementary.pdf]

Table S1. Components of the HEI-NVS and their standards for scoring (modified from [1])

Component	Max. points	Standard for max. point value[1]
Fruits	10 (5 additional points possible)	≥ 250 g/day (2 servings)
Vegetables	10 (5 additional points possible)	≥ 400 g/day (3 servings)
Beverages	10	≥ 1.5 l/day
Grains, grain products, potatoes	10	350-560 g/day
Milk, dairy products incl. cheese	10	2 servings/day[2]
Fish	10	150-220 g/week
Meat, meat products, processed meat	10	< 300-600 g/week
Eggs	10	≤ 3 /week
Alcohol	10	≤ 10 g ethanol/day (women[3])
Spreadable fats	10	≤ 15-30 g/day

[1]based on a healthy adult with a physical activity level (PAL) of 1.4; [2]Portion sizes: 200-250 g milk/yoghurt or 50-60 g cheese/fresh cheese; [3] value for non-pregnant women

Table S2. Dietary intake of women following a "good quality" or "medium quality" diet (mean ± SD).

Food category	Recommended intake	Good quality diet (*n*=41)	Medium quality diet (*n*=69)	*p*-Value*
Grains (g/day)	350-550	380.6 ± 125.5	367.0 ± 183.8	0.677
Fruits (g/day)	250	408.5 ± 212.0	319.8 ±226.0	0.044
Vegetables (g/day)	400	995.1 ± 344.9	645.7 ± 485.8	≤0.001
Milk/milk products (g/day)	100-500	664.6 ± 341.4	879.7 ± 531.0	0.022
Meat/meat products (g/week)	<300-500	876.1 ± 507.5	976.2 ± 487.0	0.307
Thereof, red meat (g/week)		602.2 ± 486.6	970.9 ± 716.1	0.004
Eggs (number/week)	<3	2.0 ± 1.0	2.1 ± 1.6	0.751
Fish (g/week)	150-220	230.5 ± 157.7	232.6 ± 312.5	0.968
Spreadable fats (g/day)	<15-30	21.8 ± 18.1	39.8 ± 31.7	0.001
Beverages (ml/week)	>1,500 ml	2,931.7 ± 1,112.3	2,936.2 ± 1,299.3	0.986

*T-test for 2-group comparisons, $p \leq 0.05$ was defined as level of significance

Table S3. Comparison of the Healthy Eating Index (HEI, total and subcategories) by GWG groups (mean ± SD)

Food category	Low-GWG (*n*=16)	Normal-GWG (*n*=40)	High-GWG (*n*=53)	*p*-Value*
HEI, total	81.3 ± 11.6	82.1 ± 10.9	85.1 ± 11.9	0.412
HEI grains	8.2 ± 1.9	8.3 ± 1.9	8.6 ± 1.8	0.140
HEI milk/milk products	5.4 ± 2.2	5.3 ± 2.8	5.0 ± 2.6	0.452
HEI fruits	11.5 ± 4.2	10.0 ± 4.5	12.1 ± 3.6[a]	0.420
HEI vegetables	12.0 ± 4.0	11.9 ± 4.3	12.7 ± 3.5	0.792
HEI fish	3.9 ± 4.0	5.2 ± 3.8	5.2 ± 4.3	0.465
HEI meat/meat products	7.0 ± 2.1	6.7 ± 2.5	7.2 ± 2.4	0.086
HEI eggs	9.3 ± 1.4	9.4 ± 1.4	9.5 ± 1.1	0.878
HEI alcohol	10.0 ± 0.0	10.0 ± 0.0	10.0 ± 0.0	[b]
HEI spreadable fats	7.8 ± 2.9	8.3 ± 2.6	8.4 ± 2.2	0.533
HEI beverages	10.0 ± 0.0	10.0 ± 0.2	9.8 ± 1.2	0.825

*Chi² test for intergroup comparisons; $p \leq 0.05$ was defined as level of significance
[a] $p = 0.015$ vs Normal-GWG (t-test for 2-groups comparison); [b] could not be calculated (constant variable); Legend: BMI, Body Mass Index; GWG, gestational weight gain

Table S4. Comparison of the Healthy Eating Index (HEI, total and subcategories) by pre-pregnancy BMI class (mean ± SD)

**Food category**	**BMI< 30 kg/m²** (*n*=93)	**BMI≥30 kg/m²** (*n*=17)	***p*-Value***
HEI, total	84.5 ± 11.3	77.8 ± 11.5	0.027
HEI grains	8.36 ± 1.9	8.6 ± 2.0	0.597
HEI milk/milk products	5.3 ± 2.7	4.3 ± 1.7	0.136
HEI fruits	11.5 ± 3.9	9.6 ± 5.0	0.068
HEI vegetables	12.3 ± 3.9	12.4 ± 4.1	0.973
HEI fish	5.4 ± 4.1	2.9 ± 3.4	0.020
HEI meat/meat products	7.07 ± 2.4	6.4 ± 2.4	0.290
HEI eggs	9.4 ± 1.2	9.5 ± 1.4	0.826
HEI alcohol	10.0 ± 0.0	10.0 ± 0.0	[a]
HEI spreadable fats	8.3 ± 2.3	8.2 ± 3.03	0.888
HEI beverages	10.0 ± 0.2	9.4± 2.1	0.009

*t-test for 2-groups comparisons, $p \leq 0.05$ was defined as level of significance; [a] could not be calculated, as the SDs of both groups were equal; Legend: BMI, Body Mass Index

Table S5. Comparison of the Healthy Eating Index (HEI, total and subcategories) by age group (mean ± SD)

**Food category**	**Age: 18-34.9 years** (*n*=64)	**Age ≥ 35 years** (*n*=46)	***p*-Value***
HEI, total	80.7 ± 11.5	87.3 ± 10.5	0.002
HEI grains	8.4 ± 2.0	8.5 ± 1.7	0.754
HEI milk/milk products	4.8 ± 2.8	5.6 ± 2.2	0.129
HEI fruits	10.9 ± 4.5	11.7 ± 3.5	0.324
HEI vegetables	11.4 ± 4.4	13.6 ± 2.7	0.004
HEI fish	4.8 ± 4.1	5.3 ± 3.9	0.498
HEI meat/meat products	7.0 ± 2.5	7.0 ± 2.2	0.915
HEI eggs	9,4 ± 1.4	9.5 ± 1.0	0.480
HEI alcohol	10.0 ± 0.0	10.0 ± 0.0	[a]
HEI spreadable fats	8.1 ± 2.6	8.6 ± 2.2	0.263
HEI beverages	9.8 ± 1.1	10.0 ± 0.2	0.437

*t-test for 2-groups comparison, $p \leq 0.05$ was defined as level of significance; [a] could not be calculated, as the SDs of both groups were equal

REFERENCES

1. Hoffmann, I.; Spiller, A.; (Hrsg.). Auswertung der Daten der Nationalen Verzehrsstudie II (NVS II): eine integrierte verhaltens- und lebensstilbasierte Analyse des Bio-Konsums. https://orgprints.org/id/eprint/18055/1/18055-08OE056_08OE069-MRI_uni-goettingen-hoffmann_spiller-2010-verzehrsstudie.pdf (Accessed October 2, 2021).
